# Dementia in Africa: progress, priorities, and emerging issues

**DOI:** 10.1002/alz.70956

**Published:** 2025-12-09

**Authors:** Adesola Ogunniyi, Mahmoud Maina, Valentine Ucheagwu, Tracey Naledi

**Affiliations:** ^1^ College of Medicine University of Ibadan Ibadan Nigeria; ^2^ Biomedical Science Research and Training Centre (BioRTC) Yobe State University Damaturu Nigeria; ^3^ Sussex Neuroscience School of Life Sciences University of Sussex Brighton UK; ^4^ Department of Psychology Nnamdi Azikiwe University Awka Nigeria; ^5^ Cognitive Research Lab Nnamdi Azikiwe University Awka Nigeria; ^6^ Department of Psychiatry the University of Michigan Ann Arbor USA; ^7^ Public Health Medicine UCT Cape Town South Africa

According to Global Burden of Diseases estimates, Africa is projected to become a hotspot of a very significant dementia burden in the coming decades because of the escalating rates of cases, with a percentage increase of over 300% in most African regions.^1^ This trend is largely attributed to the population living longer, but changing lifestyles with a rising prevalence of modifiable vascular risk factors are also a contributing factor. This is quite worrisome because of the enormous cost of care necessary for looking after those affected, which is beyond the capacity of most countries in Africa and changing family dynamics due to the dismantling of the extended family system that previously served as a buffer for care provision.

Publications on dementia in Africa started in 1983, but the overall quantity is small relative to other regions of the world, and the scope has been largely on the epidemiology of dementia, initially from a handful of countries (South Africa, Nigeria, Egypt, and Kenya). According to a review by Akinyemi et al., as of 2021, the number of countries with publications on dementia had grown from four to almost 20 with documentations on prevalence estimates, incidence rates, mortality, and risk factors.^2^ Of special interest was the lower risk of Alzheimer's disease (AD) in Africans possessing the homozygous ɛ4 allele of apolipoprotein E (APOE), the most important risk factor for sporadic, late‐onset disease in individuals of Western origin, and with the highest risk in Japanese people. This has provided a unique opportunity to search for protective haplotypes from genome‐wide association studies.^3^ Although the present tally of publications has surpassed 1000 with wider geographic coverage of almost all African countries, studies on cognition have predominated. Compared with Latin America and Southeast Asia, studies on neuroimaging, genetics, and fluid biomarkers, which are essential for accurate diagnosis, are very limited,^4^ a reflection of a scarcity of research facilities in Africa. Variations in study methodology have also made comparison of burden of disease estimates between countries difficult, some of which may reflect a possible environmental influence. There are, thus, tremendous opportunities for research on dementia in Africa with its unique demographic, social, and health systems contexts.

This special issue of the journal is dedicated to showcasing Africa's research efforts in dementia to ensure its visibility to policymakers, health system actors, and the community. Twenty‐one original research articles that passed the rigorous editorial process are being featured. Ten of them provide epidemiological data (screening, risk factors, and Knowledge, Attitude Practice data), five are on plasma biomarkers and biobanking, three highlight information on social capital and health economics, and the last three cover traditional medical practice, caregiving, and basic science. Overall, this is considered a good mix, and the selection shows the variety as well as unique features of dementia in Africa, although there was no manuscript on the current use of anti‐amyloid therapy, which is in vogue in Western countries. We consider these articles the tip of the iceberg as more publications are expected from the Africa Dementia Consortium, the AFRICA‐FINGERS, the Health and Aging in Africa: A Longitudinal Study in South Africa (HAALSI) project, and the Strengthening Responses to Dementia in Developing Countries studies (STRIDE). African researchers are thus making haste slowly in catching up with the rest of the world to combat the looming epidemic of dementia, and their studies will bring enhanced visibility to the African context.

The Alzheimer's Association (AA) deserves praise for supporting research efforts in Africa. This special issue provides a unique opportunity for African researchers in the dementia space to gain the right traction and invite collaborations to better understand the rich genetic diversity and hitherto unexplored cultural factors that may be important in preventive strategies for treating dementia. Inclusion of data from African studies will guarantee a global approach to addressing dementia, one that integrates insights from functional genomics alongside health systems and social, cultural, and structural determinants that shape health outcomes. Such knowledge can then be used to investigate mechanisms for the development of molecular therapeutic targets as well as non‐pharmacological interventions while also informing strategies that strengthen social and cultural resilience and promote inclusive, robust health systems.

## CONFLICT OF INTEREST STATEMENT

None of the authors has any conflicts of interest. Author disclosures are available in the supporting information.

## Supporting information



Supporting Information
